# Sarcasm use in Turkish: The roles of personality, age, gender, and self-esteem

**DOI:** 10.1371/journal.pone.0276073

**Published:** 2022-11-10

**Authors:** Natalia Banasik-Jemielniak, Piotr Kałowski, Büşra Akkaya, Aleksandra Siemieniuk, Yasemin Abayhan, Duygu Kandemirci-Bayız, Ewa Dryll, Katarzyna Branowska, Anna Olechowska, Melanie Glenwright, Maria Zajączkowska, Magdalena Rowicka, Penny M. Pexman

**Affiliations:** 1 Institute of Psychology, The Maria Grzegorzewska University, Warsaw, Poland; 2 Faculty of Psychology, University of Economics and Human Sciences, Warsaw, Poland; 3 Ankara Yıldırım Beyazıt University, Ankara, Turkey; 4 Faculty of Polish Studies, University of Warsaw, Warsaw, Poland; 5 Psychology Department, Hacettepe University, Ankara, Turkey; 6 Ege University, İzmir, Turkey; 7 Faculty of Psychology, University of Warsaw, Warsaw, Poland; 8 Department of Psychology, University of Manitoba, Manitoba, Canada; 9 Department of Psychology, University of Calgary, Alberta, Canada; University of Padova, ITALY

## Abstract

This study examined how self-reported sarcasm use is related to individual differences in non-Western adults. A sample of 329 Turkish speakers of high socioeconomic status completed an online survey including measures of self-reported sarcasm use, personality traits, positive and negative affect, self-presentation styles, self-esteem, as well as age and gender. Participants who reported being more likely to use sarcasm in social situations had scores indicating that they were less agreeable, less conscientious, and less emotional stable (i.e., more neurotic). Also, those who reported using sarcasm more often tended to be younger and had lower self-esteem. Self-reported sarcasm use was also positively related to both the self-promoting and the self-depreciating presentation styles. In addition to highlighting the complex relationship between individual differences and language production, these findings underscore the importance of expanding sarcasm research to include non-Western samples.

## Introduction

A significant part of everyday verbal communication involves nonliteral language, including sarcasm [1] Sarcasm is a type of figurative language used to convey criticism (and less commonly compliments) indirectly, and often in a humorous fashion [2, 3]. An example of sarcasm would be saying “*I never would have imagined that*!” to someone who has just said something obvious. Here, the intended nonliteral meaning is the reverse of the literal meaning. Despite the differences between various theoretical accounts, this contrast or duality is assumed to be the central mechanism of sarcasm and of its pragmatic effects of humor and criticism [2, 4, 5]. However, sarcasm has been variously defined in relation to *verbal irony;* either as interchangeable, related, where sarcasm is described as a type of verbal irony, or as distinct (see, e.g., [6]). Our study concerned sarcasm, and we consistently use the label sarcasm. However, for reasons of accuracy, when discussing other studies in this paper, we will use the terms sarcasm/irony according to the original authors’ assumed terminology.

The literature on sarcasm and verbal irony [5, 7–9] tends to emphasize comprehension rather than production and focuses on related linguistic and discourse factors. Research on sarcasm use is limited and not much is known about the individual characteristics of the speaker which may be related to their sarcasm use. Thus far, studies have examined a few such individual characteristics, including gender [10–12], knowledge of cultural norms [13], or second language proficiency [14]. Some personality traits have also been shown to be related to sarcasm use, such as anxiety [12] or trait anger [15–17]. Importantly, there are still few such studies, and they usually examine single factors in isolation. Other individual differences in personality, self-esteem, or self-presentation style might also play a role [15, 18]. Moreover, most studies on the individual differences in sarcasm comprehension, appreciation or production have been conducted in English with English-speaking participants, thereby raising the question of generalizability. Thus, in our research, we decided to include individual factors that might be related to sarcasm production such as the personality of the speaker, gender and age, self-esteem, self-presentation styles, and positive and negative affect. Additionally, we examined these variables in a Turkish sample.

### Sarcasm use in Turkish

In the Turkish language, the concept of sarcasm and irony seems to be very rich semantically. There are several relevant Turkish words, most of which reflect a similar but not a complete or exact meaning of irony and sarcasm. For example, the word sarcasm does not have a definite equivalent in Turkish language. It is often translated with words meaning scoff, allusion, and ridicule. Even though some words derived from Turkish (e.g., alay, iğneleme) and Arabic (e.g., kinaye, tariz) address the meaning of sarcasm partially, none of these words exactly match the meaning (https://sozluk.gov.tr/). Despite the ambiguity of the term, the use of sarcasm in Turkish is frequent. The use of sarcasm as a literary form can most commonly be seen as “Hiciv” in Turkish literature, which is used to scoff, criticize or satirize. Examples of sarcasm can be seen in a wide range of artworks, from Turkish folk art to high literature, including poems, songs, epic poems, and so forth [19]. Notably, the psycholinguistic literature on sarcasm use in Turkish is sparse and so far, no study has employed quantitative methods to assess the relationship between individual factors and sarcasm use specifically in the Turkish context.

#### Personality of the sarcastic speaker

The tendency toward sarcasm production can systematically vary between individuals, depending on their personality traits [15, 16, 20]. Bruntsch and Ruch [16] found that psychoticism correlated significantly with sarcasm (referred to as “irony” in the original study), whereas neuroticism and extraversion did not. Bruntsch and Ruch [20] also considered some other personality-related dispositions of the sarcastic speaker and found that sarcasm use was related to an inclination to break with social conventions and to use aggressive humor. Several other studies have shown that irony use is related to single personality traits: anxiety [12], and trait anger [17]. Yet the traits examined so far in relation to sarcasm use are not exhaustive, and there might be some other influential personality factors, or combinations of factors to consider.

#### Gender and age

Regarding gender, existing research has shown that men generally report more frequent sarcasm use and more positive interpretations of sarcastic utterances [10, 11, 12, 21]. The type of conversational dyad might also diversify the frequency and type of sarcastic utterances used. For instance, Lampert and Ervin-Tripp [21] found that conversational turns in male-male dyads are characterized as more teasing, but in mixed-gender conversations, men prefer self-deprecating jokes while women value more teasing ones. In addition, men rate themselves as more likely to use sarcasm than do women [22, 23], evaluate sarcasm as funnier than women, who more often report it as critical [23], are perceived as more sarcastic than women, by both genders [24], and use sarcasm more often in conversations [1, 10]. Thus, gender can influence one’s attitude towards, and tendency to use, sarcasm in daily conversations.

Gender differences in sarcasm use and understanding might also be related to broader cultural differences, norms, and expectations regarding communication, involving such cultural dimensions as masculinity versus femininity or uncertainty avoidance. For example, a Polish study by Hornowska and Charytonik [25] found that men reported using the aggressive humor style—also comprising sarcasm—more often than did women, but this result was not consistent with Kazarian and Martin’s study [26] in an Armenian-Lebanese sample nor with Chen and Martin’s findings [27] in a Chinese sample. However, a Turkish study by Başak and Can [28] showed that men reported using both aggressive and self-defeating humor styles. Therefore, examining gender differences in sarcasm use in a non-Western context, in particular, seems warranted.

Further, while age has not been shown to significantly influence sarcasm use and understanding beyond broad childhood developmental stages [29, 30], Ruch et al. [31] showed that irony, sarcasm, and cynicism were more popular among younger than older people. However, the Ruch et al. [31] study was conducted on English-speaking samples, and thus, generalizing these outcomes to different populations might be inaccurate. We hypothesized that age would also be an important factor for sarcasm use and that age would correlate negatively with self-reported sarcasm use.

#### Self-esteem

Self-esteem is defined as a sense of personal value and self-worth, a concept referring to how a person feels about themselves [32], and an individual’s subjective evaluation of their worth as a person [33]. Self-esteem was found to be related to positive emotion and prosocial behavior [32, 34]. Although the role of self-esteem in sarcasm use has not been studied directly before, it is relevant to consider the existing research on humor styles and self-esteem, as humor is a primary function of sarcasm [2, 5]. For example, Leist and Müller [35] found that the aggressive humor style (which is most commonly assumed to comprise sarcasm, see [36]) is the only humor style not correlated with self-esteem, either positively or negatively. Yue et al. [37] similarly found a positive relationship between self-esteem and affiliative and self-enhancing humor styles, but not the aggressive humor style. On the other hand, Zhao et al. [38] reported a negative correlation between self-esteem and the aggressive humor style in a sample of Chinese students. Finally, Vaughan et al. [39] found that “individuals with stable high self-esteem reported the highest levels of affiliative humor as well as the lowest levels of aggressive and self-defeating humor” (p. 309). However, though the aggressive humor style is typically associated with sarcasm, it may not fully comprise all its forms and functions, especially the affiliative ones. Indeed, Heintz and Ruch [40] have shown that scales measuring irony and sarcasm are positively correlated with all four humor styles. Furthermore, while the mocking function of sarcasm is acknowledged, a range of studies also indicates that sarcasm is perceived as funny and endearing [41–44]. Thus, based on the above, we adopted a broader perspective and hypothesized that self-esteem would be positively related to sarcasm use such that people with low self-esteem will tend to avoid using sarcasm to avoid potential ambiguity and misunderstandings, while people with high self-esteem will use sarcasm more frequently to capitalize on its pragmatic flexibility and capacity for humor.

#### Self-presentation

Self-presentation style is defined by Wojciszke [45] as actions undertaken by individuals to modify the way they are perceived by others. One of the functions of self-presentation styles is to humorously shape everyday interactions [46]. Such humor-related behaviors might also be associated with sarcasm use. Bruntsch and Ruch [16] found that the histrionic self-presentation style, involving the tendency to draw attention to oneself by imitating, role-playing, or acting [46], correlates positively with sarcasm use. Mendiburo-Seguel and Heintz [47] also reported a positive correlation between sarcasm use and the humorous self-image. However, not much is known about the general relationship between self-presentation styles and sarcasm use. Expanding the results of Bruntsch and Ruch [16], suggesting that the willingness to draw attention to oneself might be associated with higher sarcasm use, one could expect that both self-promoting and self-depreciating presentation styles may be related to higher sarcasm use.

#### Positive and negative affect

Sarcasm can be used in both negative and positive contexts, to either criticize (e.g., “A fine friend you are!” said to a friend who failed to be helpful) or praise (e.g., “You’re a terrible friend!” said to a friend who has been extremely helpful; [48]). However, sarcasm is prototypically negative, even if it can be used for affiliative functions, to build group solidarity, or to praise humorously. Sarcasm is considered to always have an element of negative evaluation, and its use is often related to verbal aggression [49]. For this reason, it could be expected that a high extent to which one generally experiences negative emotions such as nervousness, sadness, and irritation may be related to higher sarcasm use, either for the purposes of expressing these negative emotions or as a coping strategy [50].

Finally, it is worth noting that, with some exceptions (see [1, 21]), most of the studies described above used questionnaire-based self-report measures of sarcasm use. For example, sarcasm is most commonly identified with the aggressive humor style in Martin et al.’s [36] Humor Styles Questionnaire. Ruch et al. [31] have also developed a questionnaire which measures subjective preferences towards using sarcasm and irony, understood as separate forms of humor. Other studies (see, e.g., [11, 16]) have used experimental tasks in which participants rated their likelihood of using sarcasm or chose a response option from a pre-designed set in a situation depicted in the stimulus materials. Therefore, while studies using naturalistic data (e.g., recordings of everyday or experimentally arranged conversations) are warranted, studies on the psycholinguistics of sarcasm typically use questionnaire- and rating-based measures. The present study continues this tradition.

#### The present study

Thus, in the present study, we investigated self-reported sarcasm use and its relationships with gender, age, Big Five personality traits, positive/negative affect, self-esteem, and self-presentation style in a Turkish sample. We also explored the predictive power of these variables in analysis of sarcasm use. Our hypotheses, based on the previous studies where possible, are listed below.

H1 Agreeableness and conscientiousness will correlate negatively with self-reported sarcasm use, whereas neuroticism, openness to experience, and extraversion will correlate positively with self-reported sarcasm use.

H2 Age and self-reported sarcasm use will be negatively correlated.

H3 Men will report using sarcasm more often than women.

H4 Self-presentation styles will be positively correlated with self-reported sarcasm use.

H5 There will be a positive correlation between sarcasm use and self-esteem.

H6 There will be relationships between positive and negative affect and sarcasm use.

**Fig 1:**
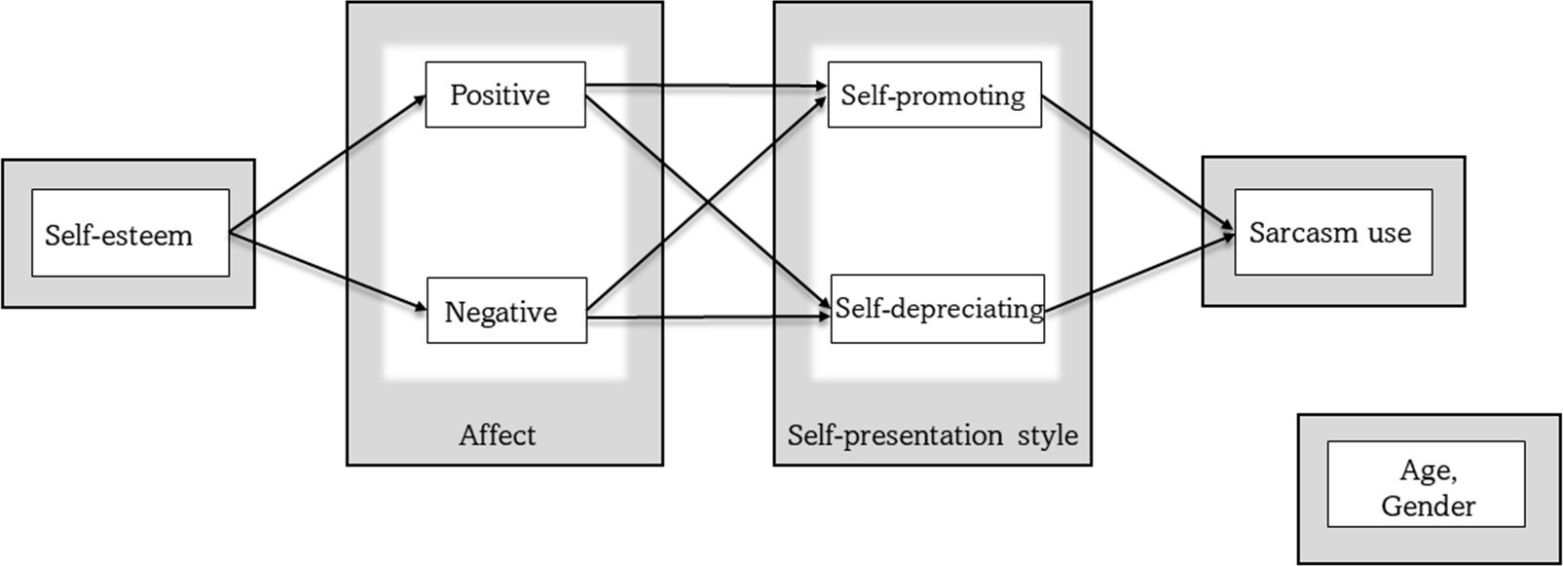
The proposed model.

We first conducted a series of correlational analyses. Next, to further explore the relations between sarcasm use and individual factors, we carried out structural equation modeling (SEM), based on the theoretical model presented in Fig 1, which is described in the Results section.

## Method

### Participants

A total of 351 participants completed the study. Informed consent in written form (submitted online) was obtained from all participants before data collection. The data from 22 participants were removed: six participants gave incomplete or inappropriate responses; four participants were under the age of 18; eleven participants reported that their native language was other than Turkish, and one person described themselves as bilingual. Therefore, the final sample was *N* = 329, including 250 women and 79 men (*M*_age_ = 37 years, range: 18–70 years). Women were coded as 1, men were coded as 0. Two hundred and eighty-five participants had higher education (university degree). Forty-four participants had secondary education or lower. Participants were recruited from two Turkish universities in large-sized cities and through social media. While this method of data collection allowed us to effectively reach a sample of appropriate size, the participants with university degrees were over-represented, relative to the general population in Turkey. This point is elaborated in the Limitations section below.

### Materials

To measure self-reported sarcasm use, we translated *The Sarcasm Self-Report Scale* (SSS, [22]) into Turkish using the back-translation method [51]. The SSS is a 16-item questionnaire measuring the self-reported tendency to use sarcasm. Items are answered on a 7-point scale (1—*not at all likely*; 7—*extremely likely*). Example items are “How sarcastic do you think you are?” and “How often do you make sarcastic statements during daily interactions?” The SSS was scored by computing a sum of all item answers. In this study, the Cronbach’s α reliability coefficient for the SSS was .92, which is comparable with that reported in the original study by Ivanko et al. [22]. The results of the original study by Ivanko et al. [22] provided validation for the SSS by showing that the participants’ SSS scores were a significant predictor of sarcasm use in real conversations. To check the construct validity of the Turkish version of the SSS, we investigated the relationship between the Turkish participants’ SSS scores and their scores on the aggressive humor subscale of the Humor Styles Questionnaire (HSQ, [36]). To this end, we used a published Turkish adaptation of the HSQ [52]. According to previous research on sarcasm, it is related to the four humor styles [40]. The results showed a significant positive correlation between aggressive humor style scores and SSS general scores (*r* = .52, *p* = .01), and between self-defeating humor scores and general SSS scores (*r* = .40, *p* = .01). Thus, these relatively large and robust correlations serve as a preliminary validation of the Turkish version of the SSS.

*The Ten-Item Personality Inventory* (TIPI, [53]) in a Turkish adaptation by Atak [54] was used to measure the Big Five traits of extraversion, agreeableness, conscientiousness, emotional stability (neuroticism), and openness to experience (two items for each factor). Answers were given on a 7-point scale (1—*disagree strongly*; 7—*agree strongly*). Example items include “I see myself as critical, quarrelsome” and “I see myself as sympathetic, warm.” The test was scored by reverse-scoring appropriate items and averaging the two items per each Big Five trait. The Cronbach’s α reliability coefficient in the Turkish adaptation study by Atak [54] was .83 for openness to experience, .81 for agreeableness, .83 emotional stability, .84 for conscientiousness, and .86 for extraversion. In the current sample, reliabilities were as follows: .16 for agreeableness, .49 for emotional stability, .39 for openness to experiences, .49 for conscientiousness, and .52 for extraversion. Therefore, due to the unsatisfactory reliability coefficients, this measure was not included in any further analyses in the current study.

*The Positive and Negative Affect Scale* (PANAS, [55]) in a Turkish adaptation by Gençöz [56] was used. The 20-item PANAS measures the respondent’s current positive (e.g., excited, enthusiastic, active) and negative (e.g., upset, guilty, nervous) affect (10 items for positive and negative affect, respectively). Answers are given on a five-point scale (1—*very slightly or not at all*; 5—*extremely*). The test was scored by computing a sum of 10 items for the positive affect subscale and 10 items for the negative affect subscale. The Cronbach’s α reliability coefficients in the original Turkish adaptation study [56] were .83 and .86 for the positive and negative affect subscales. In this study, they were .89 and .87, respectively.

*The Rosenberg Self-Esteem Scale* (RSES, [57]) in a Turkish adaptation by Tukuş [58] was used to measure self-esteem, defined as the subjective evaluation of one’s overall worth as a human being. Answers to the 10-item scale are given on a four-point scale (1—*strongly disagree*; 4—*strongly agree*). Example items are “I wish I could have more respect for myself” and “I feel that I have a number of good qualities.” The RSES was scored by calculating a sum of all items once the relevant items had been reverse-coded. The Cronbach’s α reliability coefficient in the Turkish adaptation study by Tukuş [58] was .90 for the whole scale. In the current sample, Cronbach’s α coefficient was .88.

*The Self-Presentation Styles Questionnaire* (SSQ, [45]), back-translated into Turkish for the purposes of this study, was used to measure self-presentation, understood as the behaviors undertaken to create a certain impression about oneself in others. The 30-item SPSQ measures *self-promotion* (positive, competent, reliable) and *self-depreciation* (negative, incompetent, but also humble) styles. Answers are given on a five-point Likert-type scale (1—*never*; 5—*very often*). Example items are “I avoid talking about my successes” and “I speak in a decisive tone, even when I am not certain.” The SPSQ was scored by computing a sum of 15 items in the self-promotion subscale and 15 items in the self-depreciation subscale. Cronbach’s α values for the original measure by Wojciszke [45] for the subscales of self-promotion and self-depreciation were .87 and .82, respectively. In this study, they were .86 and .80, respectively.

With the exception of the SSQ, which the current study did not attempt to validate, the Turkish language versions of the measures other than the SSS were taken from previously published studies carried out on Turkish samples and showing their satisfactory validity.

We also included a series of questions about demographic data, that is, gender, age, socioeconomic status (i.e., level of education and occupation), place of residence, and bilingual/multilingual status.

### Procedure

We collected data by distributing a link to an online survey on social media and shared the link with students of two Turkish universities, asking for their voluntary participation. The study was performed online using Google Forms. After being acquainted with the general purpose and quantitative character of the study, and the anonymity of the responses and the option to withdraw participation at any point, the participants provided their informed consent (by ticking an appropriate checkbox). Then, the participants filled out the PANAS, the TIPI, the SSS, the SPS, the RSES, and the demographic questionnaire. At the end, the participants were presented with more detailed information about the study. The procedure was approved by the Ethics Committee of [AUTHOR_1 AFFILIATION].

## Results

By comparing the relations of personality factors, age, gender, self-esteem, and self-presentation styles with self-reported sarcasm use in a Turkish sample, we hoped to provide insights about individual differences that would facilitate future cross-cultural studies on sarcasm. As the reliability coefficients for TIPI were unsatisfactory (ranging from .15 for agreeableness to .51 for extraversion), we did not include this variable in any further analyses.

We examined whether the variables deviated from the normal distribution using the values of skewness and kurtosis, and by analyzing the histograms. Based on Kim’s [59] recommendations for large samples (above 300), we concluded that the variables investigated in the current study did not deviate from the normal distribution (absolute values of skewness and kurtosis did not exceed 1, S1 Table).

### Sarcasm use and gender

First, we explored the relation between self-reported sarcasm use and gender (H3). An independent groups t-test revealed no statistically significant differences in self-reported sarcasm use between women (*M* = 47.2, *SD* = 18.22) and men (*M* = 51.4, *SD* = 21.53), *t* = 1.558, *p* = .122. Thus, H3 was not confirmed.

### Correlational analyses

#### Sarcasm use and age

To test our hypothesis on the relation between self-reported sarcasm use and age (H2), we ran a correlational analysis using Pearson’s *r*. Age showed a moderate, negative correlation with self-reported sarcasm use, thereby indicating that older people tend to judge that they are less likely to use sarcasm than younger people, *r* (327) = -.398, *p* < .001. H2 was thus confirmed. The correlation coefficients are presented in Table 2.

#### Sarcasm use, self-esteem, and self-presentation styles

Our H5 was that self-esteem would be positively correlated with self-reported sarcasm use. A statistically significant negative correlation indicated that people with lower self-esteem also tended to report a greater tendency to use sarcasm, *r* (327) = -.170, *p* < .001. This result did not confirm H5, which anticipated a positive correlation.

To test our H4 on the relation of self-reported sarcasm use and self-presentation style, we ran additional correlations. We found that the SSS score was positively correlated with both self-presentation styles, *r* (327) = .424, *p* < .001, and *r* (327) = .235, *p* < .001 for *self-promotion* and *self-depreciation*, respectively. This indicates that people who tended to present themselves as competent and people who presented themselves as modest tended to report using sarcasm more often than those who reported lower tendencies to use self-promoting and self-depreciating self-presentation styles. H4 was thus confirmed.

#### Sarcasm use and positive/negative affect

The relationships between self-reported sarcasm use and both positive and negative affect (H6) were also explored using correlation analysis. There was a positive correlation between self-reported sarcasm use and the negative affect PANAS subscale, *r* (327) = .155, *p* < .001. However, self-reported sarcasm use was unrelated to the positive affect PANAS subscale, *r* (327) = -.069, *p* = .212. The analysis shows that negative affect is related to reported sarcasm use; that is, the higher the tendency to feel negative emotions such as anxiety, sadness, fear, anger, guilt, shame, and irritability, the greater the tendency to report use of sarcasm. H6 was partially confirmed. Correlation coefficients are presented in Table 1.

**Table 1:** Pearson’s correlation coefficients.

	1.	2.	3.	4.	5.	6.
1. Age	--					
2. Self-esteem	.215***	--				
3. Positive Affect	.144**	.473**	--			
4. Negative Affect	-.126*	-.442**	-.326**	--		
5. Autopresentation (self-promotion)	-.174**	.082	.103	.137*	--	
6. Autopresentation (self-depreciation)	-.203***	-.464**	-.257**	.220**	.123*	--
7. Sarcasm	-.398***	-.170**	-.069	.155**	.424**	.235**

*p < .05.

**p < .01.

***p < .001

### Predictors of self-reported sarcasm use–SEM

To further explore the indirect effects of self-esteem, positive/negative affect and self-presentation on self-reported sarcasm use, we conducted Structural Equation Modelling (SEM; [60]). We hypothesized that the relationship between self-esteem and sarcasm use will be mediated by positive/negative affect and self-presentation styles. As high self-esteem is related to positive emotions and prosocial behavior [32], we hypothesized that self-esteem would promote positive affect and inhibit negative affect, and that both positive and negative affect would influence self-presentation styles, which in turn would lead to increased sarcasm use.

**Fig 2:**
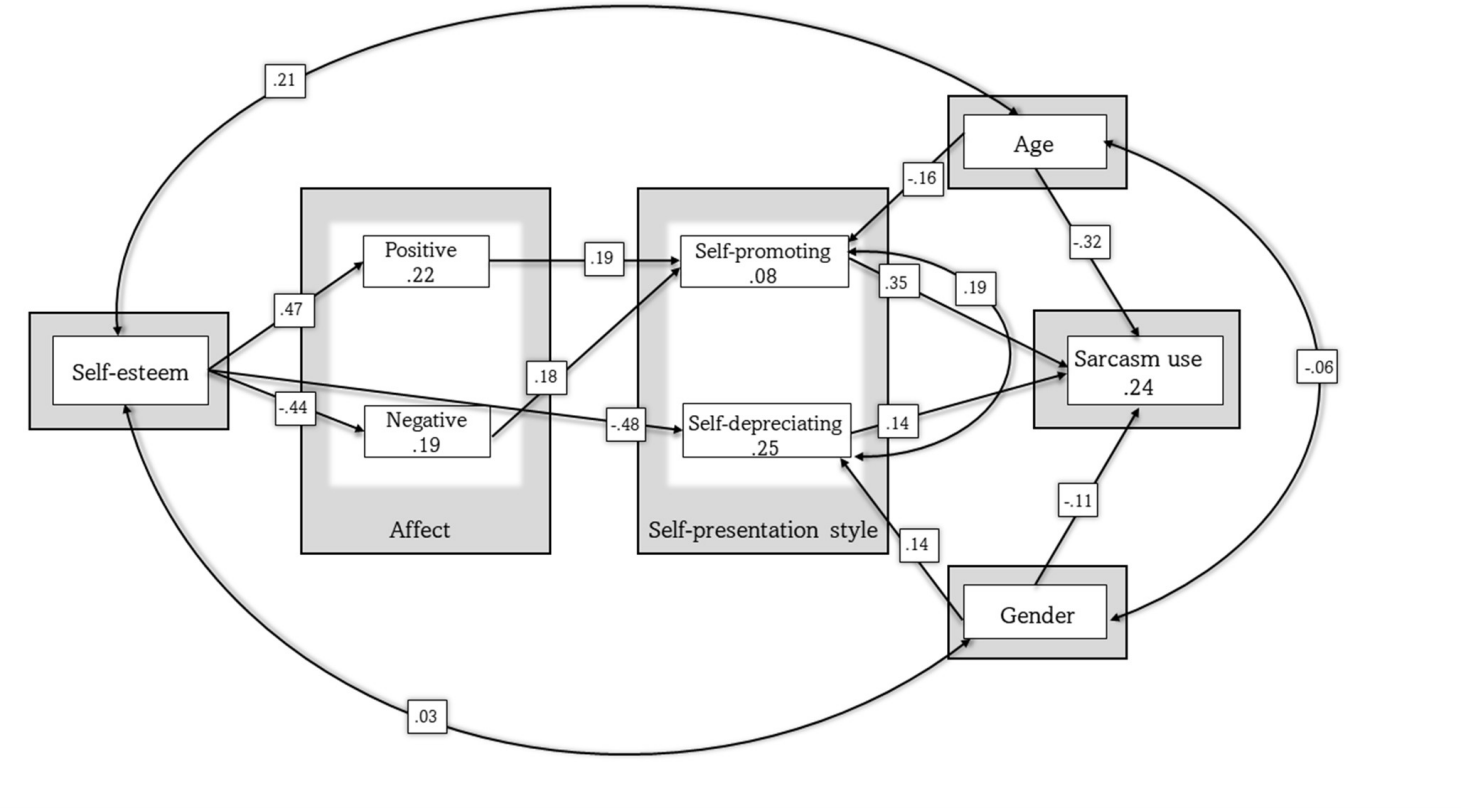
Model 5.

Our hypotheses were based on previous studies showing the relation of sarcasm to histrionic self-presentation, oriented around focusing attention on oneself [16, 46]. Positive and negative social effects of sarcastic self-mockery were highlighted by Ungar [61]. Tsukawaki and Imura [62] have also proposed separating self-deprecating humor into benign and deleterious types, which might potentially comprise self-directed, sarcastic humor. Our results obtained in the correlational analysis seem in line with these propositions. Thus, we tested whether the relationship between self-esteem and sarcasm use would be mediated by negative and positive affect, and by two self-presentation styles (see Fig 2). Additionally, we controlled for age and gender.

Model 1 performed poorly, with unsatisfactory model fit indices.

In order to improve the model, we checked the modification indices and in cases of them being 10 or above, we considered adding a path [63]. The modification indices suggested adding a path between self-esteem and self-depreciation, revealing a possible mediation of negative affect (Model 2), adding covariance between self-promotion and self-depreciation (Model 3). We also decided to remove the statistically nonsignificant paths (from positive affect and negative affect to self-depreciation, Model 4). We decided to remove the remaining statistically nonsignificant paths (from age and gender to both positive and negative affect, and from gender to self-promotion, Model 5). Model 5 had excellent fit parameters [64]. We compared Models 4 and 5 using chi-squared difference [65], which was not statistically significant, χ2 (6) = 8.89; *p* >.05. This means that Models 4 and 5 had very similar fit parameters. The parameters for models 1 through 5 are presented in Table 2. In Table 3, we present standardized coefficients in Model 4 and Model 5.

**Table 2:** Fit indices for Models 1 to 5.

Measure	CMIN	DF	CMIN/DF	GFI	AGFI	CFI	SRMR	RMSEA	PNFI	ECVI	AIC
Model 1	76.683	7	10.955	.951	.746	.833	.067	.0174	.207	.411	134.683
Model 2	27.491	6	4.582	.980	.878	.949	.038	.104	.201	.267	87.491
Model 3	16.603	5	3.321	.987	.909	.972	.034	.087	.172	.240	78.603
Model 4	16.708	7	2.387	.987	.935	.977	.034	.065	.241	.228	74.708
Model 5	25.596	13	1.969	.981	.947	.970	.046	.054	.438	.218	71.596

*Note*. CMIN = chi-squared; df = degree of freedom; GFI = goodness-of-fit index; AGFI = adjusted goodness of fit index; CFI = comparative normed fit index; SRMR = standardized root mean square residual; RMSEA = root mean squared error of approximation; PNFI = parsimony normed fit index; ECVI = expected cross validation index; AIC = Akaike’s information criterion.

**Table 3:** Standardized coefficients in Model 4 and Model 5.

			Model 4		Model 5	
			Beta	p	Beta	p
Positive Affect	<---	Self-esteem	.467	***	.473	***
Positive Affect	<---	Age	.039	.431		
Negative Affect	<---	Age	-.033	.519		
Positive Affect	<---	Gender	-.086	.077		
Negative Affect	<---	Gender	-.006	.900		
Self-promoting	<---	Positive Affect	.184	***	.187	***
Self-promoting	<---	Negative Affect	.172	.001	.176	***
Self-promoting	<---	Age	-.181	***	-.160	.002
Self-depreciating	<---	Gender	.131	.006	.145	.002
Self-depreciating	<---	Age	-.095	.052		
Self-promoting	<---	Gender	-.049	.357		
Self-depreciating	<---	Self-esteem	-.462	***	-.482	***
Sarcasm use	<---	Self-promoting	.345	***	.348	***
Sarcasm use	<---	Self-depreciating	.142	.003	.144	.002
Sarcasm use	<---	Age	-.314	***	-.317	***
Sarcasm use	<---	Gender	-.109	.018	-.110	.018
Negative Affect	<---	Self-esteem	-.434	***	-.442	***

Note.

*** p < .001.

This indicates that the relationship between self-esteem and sarcasm use was mediated by both positive and negative affect and by the self-promoting self-presentation style. Self-esteem can lead to both positive and negative affect, which influences self-promotion, which ultimately leads to higher sarcasm use.

## Discussion

The purpose of the present study was to explore the relationships of individual differences factors with self-reported sarcasm use in a sample of Turkish participants. To our knowledge, this was the first study on this topic in the Turkish context. Additionally, we examined a broad, theoretically founded set of variables and carried out SEM analyses to attempt to more comprehensively establish their contribution to self-reported sarcasm use.

We found a negative correlation between self-reported sarcasm use and self-esteem, and a positive correlation between sarcasm use and negative, but not positive, affect. Self-reported sarcasm use was positively correlated with both self-presentation styles: self-promoting and self-depreciating. The results also showed a negative correlation between self-reported sarcasm use and age. Previous studies reported that aggressive humor, which comprises irony, is either negatively related, or not statistically significantly related with self-esteem [35, 37, 38]. Using a measure of sarcasm use specifically, we confirmed this relationship. However, due to the inherently risky nature of irony [10], it is difficult to ascertain from correlations whether sarcasm use is the cause or the effect of self-esteem or negative affect (see [66]). Thus, in order to further explore the observed relationships, we conducted a SEM analysis controlling for age and gender. We obtained a model that explained significant variance in sarcasm use. The model showed that the relationship between self-esteem and sarcasm use is mediated by both positive and negative affect and by the self-promoting self-presentation style. In Model 5, we found that self-esteem can lead to both positive and negative affect, which influences self-promotion, which ultimately leads to higher use of sarcasm. Using a measure that did not specify the form of sarcasm, but rather the contexts of its use (i.e., the likelihood of using sarcasm in a given situation) together with carrying out a SEM analysis, we are able to add evidence to the claim that sarcasm can be used for both affiliative and disaffiliative as well as humorous and critical pragmatic goals [2, 41]. Previous studies have frequently examined whether sarcasm is uniformly more humorous or critical than literal speech, or examined correlations between sarcasm use and single variables [41, 43, 67, 68]. Our results suggest that traits of both a positive and negative valence (e.g., high and low self-esteem, positive and negative affect, self-enhancing and self-depreciating self-presentation style) can variously contribute to the expression of sarcasm. It may seem that traits of positive valence dominate in the model. However, self-presentation is a complex mechanism. For instance, the author of the self-presentation style scale we used found that self-promotion is positively correlated to rivalry, narcissism, competence, self-ascription, cultural masculinity, and need of achievement, whereas self-deprecation showed positive correlations with self-ascription of moral traits and a chronic tendency to complain, and negative correlations with self-esteem, narcissism, and cultural masculinity [45]. This may have interesting implications for establishing the personality correlates and predictors of sarcasm use, which is a valuable and novel research direction (see [15, 69]). For example, it may be worthwhile to consider the role of such variables as trait anger, trait narcissism, or neuroticism in greater detail. However, future studies could also consider collecting observational or task-based data rather than self-report data only in order to examine variations in the form of sarcasm driven by various individual differences. For example, Tortoriello et al. [70] showed that higher levels of Dark Triad traits influence the perception that sarcasm is helpful when giving feedback. Furthermore, since research on sarcasm use has predominantly focused on English-speaking samples, the current study, carried out on a Turkish sample, represents a more inclusive approach wherein data collection is not limited to the so-called Western, educated, industrialized, rich and democratic (WEIRD) populations. We confirmed the negative correlation between self-reported sarcasm use and self-esteem that has also been reported in German, Hong Kong, and Chinese samples [35, 37, 38]. In the current Turkish sample, age was also a negative correlate, similar to a Swiss-German study by Ruch et al. [31]. However, in contrast to several previous European and American studies [1, 10, 12, 23], we did not find statistically significant gender differences in self-reported sarcasm use. This may be due to cultural factors. Likewise, it is possible that the results of our SEM analysis were influenced by the Turkish cultural context. Sarcasm may function differently in other cultures, for example, ones characterized by higher social acceptance of hierarchies or greater cultural collectivism (such as the Turkish culture). For example, in a quantitative cross-cultural study, Blasko et al. [71] showed that US participants described themselves as more sarcastic than Chinese participants. However, several forms of expression similar to the Western concept of sarcasm have been identified as well, for example, the Chinese *bei-* ironization (see [72]). Therefore, both within-culture examinations and cross-cultural comparisons are warranted.

### Limitations

There are several limitations to the current study which should be considered when interpreting the results. Above all, although we obtained a satisfactory sample size, it was imbalanced with respect to gender and education—two variables which might potentially impact self-reported sarcasm use. The sample contained 250 women and 79 men. Studies have shown that women use humor, including sarcasm, less frequently than do men [16, 22] while men rate irony as funnier than do women and tend to rate themselves as more ironic than do women [23]. Other studies show that the patterns of sarcasm use are influenced by the gender composition of a given interactional dyad (same-gender vs. intergender; see [11, 12, 21]). Thus, considering the fact that gender differences in sarcasm use might be related to cultural norms of communication, further studies should more closely balance gender in the sample composition. Additionally, comparisons between self-reported sarcasm use in same-gender and intergender contexts of sarcasm use (e.g., achieved by manipulating the pronouns in questionnaire items) could yield potentially valuable results.

In addition, participants with university degrees were over-represented, constituting 87% of the current sample. This is in contrast to the national statistics for Turkey, showing that roughly 21% of young adults hold university degrees. While education and IQ have not been shown to significantly correlate with sarcasm use [16], such an imbalance in the current sample means that the results may not generalize to the wider population. In addition, in order to study the influence of national culture and/or cultural patterns of communication on sarcasm use, care should be taken to minimize the confounding influence of demographic factors. For this reason, future studies should aim to recruit samples that are more representative.

Also, while we believe that collecting data from a non-English speaking sample is an important contribution and one that points to the importance of including a variety of non-WEIRD samples, in future research it would be advisable to add a measure of cultural orientation in order to be able to draw conclusions about the possible relationships between cultural values measured on individual level and sarcasm use.

A further limitation might be the fact that the participants filled in the questionnaires in a fixed, not randomized order. Although both the fixed and random options lead to certain limitations, this is something that should be acknowledged.

Finally, although one of our research questions concerned personality, due to the shortcomings of using the TIPI as a measure of personality, we were unable to verify one of our hypotheses related to it.

### Conclusions

The results of the present study suggest that there is a relationship between self-esteem and sarcasm use, which is mediated by both positive and negative affect and self-promoting self-presentation style. Self-esteem can lead to both positive and negative affect, which influences self-promotion, which ultimately leads to higher use of sarcasm. Thus, several factors explain individual variability in sarcasm use and need to be incorporated in future theories of sarcasm use.

## Supporting information

S1 Table Skewness and kurtosis of the examined variables. (DOCX)

S2 Table Standardized regression coefficients for model 1. (DOCX)

## References

[1] Gibbs R. W. (2000). Irony in talk among friends. *Metaphor and Symbol*, 15(1–2), 5–27.

[2] Attardo S. (2000). Irony markers and functions: Towards a goal-oriented theory of irony and its processing. *Rask*, 12 *(*1*)*, 3–20.

[3] Roberts R. M., & Kreuz R. J. (1994). Why do people use figurative language? *Psychological Science*, 5, 159–163.

[4] Dynel M. (2014). Isn’t it ironic? Defining the scope of humorous irony. Humor, 27(4), 619–639.

[5] Garmendia J. (2018). *Irony*. Cambridge University Press.

[6] Taylor C. (2017). The relationship between irony and sarcasm: Insights from a first-order metalanguage investigation. *Journal of Politeness Research*, 13(2), 209–241. 10.1515/pr-2015-0037

[7] Giora R. (1997). Understanding figurative and literal language: The graded salience hypothesis. *Cognitive Linguistics*, 8, 183–206.

[8] Sperber D., & Wilson D. (1995). *Relevance*: *Communication and cognition* (2^nd^ Ed.). Blackwell.

[9] Utsumi A. (2000). Verbal irony as implicit display of ironic environment: Distinguishing ironic utterances from nonirony. *Journal of Pragmatics*, 32, 1777–1806.

[10] Colston H. L., & Lee S. Y. (2004). Gender differences in verbal irony use. *Metaphor and Symbol*, 19(4), 289–306. 10.1207/s15327868ms1904_3

[11] Milanowicz A. & Bokus B. (2020). *W krzywym zwierciadle ironii i autoironii*. *O kobietach i mężczyznach nie wprost* [The distorted lens of irony and self-mockery. A gender comparison]. Wydawnictwo Uniwersytetu Warszawskiego.

[12] Milanowicz A., Tarnowski A., & Bokus B. (2017). When sugar-coated words taste dry: The relationship between gender, anxiety, and response to irony. *Frontiers in Psychology*, 8, 2215. doi: 10.3389/fpsyg.2017.02215 29326634 PMC5742492

[13] Caffarra S., Motamed Haeri A., Michell E., & Martin C. D. (2019). When is irony influenced by communicative constraints? ERP evidence supporting interactive models. *European Journal of Neuroscience*, 50(10), 3566–3577. doi: 10.1111/ejn.14503 31282038

[14] Tiv M., Rouillard V., Vingron N., Wiebe S., & Titone D. (2019). Global second language proficiency predicts self-perceptions of general sarcasm use among bilingual adults. *Journal of Language and Social Psychology*, 38(4), 459–478.

[15] Bruntsch R., Hofmann J., & Ruch W. (2016). Virgin soil in irony research: Personality, humor, and the “sense of irony”. Translational Issues in Psychological Science, 2(1) 25–34. 10.1037/tps0000054

[16] Bruntsch R., & Ruch W. (2017a). The role of humor-related traits and broad personality dimensions in irony use. *Personality and Individual Differences*, 112, 139–143. 10.1016/j.paid.2017.03.004

[17] Szymaniak K., & Kałowski P. (2020). Trait anger and sarcasm use. *Personality and Individual Differences*, 154, 109–662.

[18] Tiv M., Deodato F., Rouillard V., Wiebe S., & Titone D. (2020). Second language experience impacts first language irony comprehension among bilingual adults. *Canadian Journal of Experimental Psychology/Revue canadienne de psychologie expérimentale*, 75(2), 126–132. doi: 10.1037/cep0000230 32940494

[19] Kurt İ. (2005). Yöresel anlatım içerisinde Hiciv sanatının uygulanması ve “Manda Yuva Yapmış Söğüt Dalına” gerçeği. Representation in music and musical representation Symposium, İstanbul Technical University, İstanbul. (6-7-8 October).

[20] Bruntsch R., & Ruch W. (2017b). Studying irony detection beyond ironic criticism: Let’s include ironic praise. *Frontiers in Psychology*, 8, 606. doi: 10.3389/fpsyg.2017.00606 28484409 PMC5399077

[21] Lampert M. D., & Ervin-Tripp S. M. (2006). Risky laughter: Teasing and self-directed joking among male and female friends. *Journal of Pragmatics*, 38(1), 51–72. 10.1016/j.pragma.2005.06.004

[22] Ivanko S. L., Pexman P. M., & Olineck K. M. (2004). How sarcastic are you? Individual differences and verbal irony. *Journal of Language and Social Psychology*, 23(3), 244–271. 10.1177/0261927X04266809

[23] Milanowicz A. (2013). Irony as a means of perception through communication channels. Emotions, attitude and IQ related to irony across gender. *Psychology of Language and Communication*, 17(2), 115–132. 10.2478/plc-2013-0008

[24] Pexman P. M. (2005). Social factors in the interpretation of verbal irony: The roles of speaker and listener characteristics. In Colston H. L. & Katz A. N. (Eds.), Figurative language comprehension: Social and cultural influences (pp. 209–232). Lawrence Erlbaum Associates.

[25] Hornowska E., & Charytonik J. (2011). Polska adaptacja kwestionariusza stylów humoru (HSQ) R. Martina, P. Puhlik-Doris, G. Larsena, J. Gray i K. Weir [Polish adaptation of the Humor Styles Questionnaire by R. Martin, P. Puhlik-Doris, G. Larsen, J. Gray, and K. Weir]. *Studia Psychologiczne*, 49(4), 5–22.

[26] Kazarian S. S., & Martin R. A. (2006). Humor styles, culture-related personality, well-being, and family adjustment among Armenians in Lebanon. *Humor*, 19(4), 405–423.

[27] Chen G. H., & Martin R. A. (2007). A comparison of humor styles, coping humor, and mental health between Chinese and Canadian university students. *Humor*, 20(3), 215–234. 10.1515/HUMOR.2007.011

[28] Başak B. E., & Can G. (2014). The relationships between humor styles, shyness and automatic thoughts among university students. *Egitim ve Bilim*, 39(174), 365–376.

[29] Banasik-Jemielniak N., & Bokus B. (2019). Children’s comprehension of irony: Studies on Polish-speaking preschoolers. *Journal of Psycholinguistic Research*, 48(5), 1217–1240. doi: 10.1007/s10936-019-09654-x 31312955 PMC6744549

[30] Banasik-Jemielniak N., Bosacki S., Mitrowska A., Wyrębek, Walters D., Wisiecka K., Copeland N. E., et al. (2020). “Wonderful! We’ve just missed the bus.”–Parental use of irony and children’s irony comprehension. *Plos One*, 15(2), e0228538. doi: 10.1371/journal.pone.0228538 32084153 PMC7034895

[31] Ruch W., Heintz S., Platt T., Wagner L., & Proyer R. T. (2018). Broadening humor: Comic styles differentially tap into temperament, character, and ability. *Frontiers in Psychology*, 9, 6. doi: 10.3389/fpsyg.2018.00006 29403416 PMC5778606

[32] Leary M. R., & MacDonald G. (2003). Individual differences in self-esteem: A review and theoretical integration.

[33] Orth U., & Robins R. W. (2022). Is high self-esteem beneficial? Revisiting a classic question. American Psychologist, 77(1), 5–17. doi: 10.1037/amp0000922 35357851 PMC9306298

[34] Taylor S. E., & Brown J. D. (1988). Illusion and well-being: A social psychological perspective on mental health. *Psychological Bulletin*, 103(2), 193. 3283814

[35] Leist A. K., & Müller D. (2013). Humor types show different patterns of self-regulation, self-esteem, and well-being. *Journal of Happiness Studies*, 14(2), 551–569.

[36] Martin R. A., Puhlik-Doris P., Larsen G., Gray J., & Weir K. (2003). Individual differences in uses of humor and their relation to psychological well-being: Development of the Humor Styles Questionnaire. *Journal of Research in Personality*, 37 *(*1*)*, 48–75. 10.1016/S0092-6566(02)00534-2

[37] Yue X. D., Liu K. W. Y., Jiang F., & Hiranandani N. A. (2014). Humor styles, self-esteem, and subjective happiness. *Psychological Reports*, 115(2), 517–525. doi: 10.2466/07.02.PR0.115c18z6 25153846

[38] Zhao J., Kong F., & Wang Y. (2012). Self-esteem and humor style as mediators of the effects of shyness on loneliness among Chinese college students. *Personality and Individual Differences*, 52(6), 686–690.

[39] Vaughan J., Zeigler-Hill V., & Arnau R. C. (2014). Self-esteem instability and humor styles: Does the stability of self-esteem influence how people use humor? *The Journal of Social Psychology*, 154(4), 299–310. doi: 10.1080/00224545.2014.896773 25154114

[40] Heintz S., & Ruch W. (2019). From four to nine styles: An update on individual differences in humor. *Personality and Individual Differences*, 141, 7–12. doi: 10.1016/j.paid.2018.12.008

[41] Dews S., & Winner E. (1995). Muting the meaning a social function of irony. *Metaphor and Symbol*, 10(1), 3–19. 10.1207/s15327868ms1001_2

[42] Gibbs R. W., Bryant G. A., & Colston H. L. (2014). Where is the humor in verbal irony? *Humor*, 27(4), 575–595. 10.1515/humor-2014-0106

[43] Leggitt J. S., & Gibbs R. W. (2000). Emotional reactions to verbal irony. *Discourse Processes*, 29(1), 1–24. 10.1207/S15326950dp2901_1

[44] Dar-Nimrod I., Ganesan A., & MacCann C. (2018). Coolness as a trait and its relations to the Big Five, self-esteem, social desirability, and action orientation. *Personality and Individual Differences*, 121, 1–6. 10.1016/j.paid.2017.09.012

[45] Wojciszke B. (2002). Autopromocja i autodeprecjacja: Kwestionariusz Stylów Autoprezentacji [Self-promotion and self-depreciation. The Self-Presentation Styles Questionnaire]. *Psychologia Jakości Życia*, 1, 145–171. http://depot.ceon.pl/handle/123456789/1907

[46] Renner K. H., Enz S., Friedel H., Merzbacher G., & Laux L. (2008). Doing as if: The histrionic self-presentation style. *Journal of Research in Personality*, 42(5), 1303–1322. 10.1016/j.jrp.2008.04.005

[47] Mendiburo-Seguel A., & Heintz S. (2020). Comic styles and their relation to the sense of humor, humor appreciation, acceptability of prejudice, humorous self-image and happiness. *Humor*, 33(3), 381–403. 10.1515/humor-2018-0151

[48] Kreuz R. J., & Glucksberg S. (1989). How to be sarcastic: The echoic reminder theory of verbal irony. *Journal of experimental psychology*: *General*, 118(4), 374.

[49] Averbeck J. M., & Hample D. (2008). Ironic message production: How and why we produce ironic messages. *Communication Monographs*, 75(4), 396–410. 10.1080/03637750802512389

[50] Banasik-Jemielniak N. (2019). Children’s exposure to irony in the first four years of their life: What we learn about the use of ironic comments by mothers from the analysis of the Providence corpus of CHILDES. *Psychology of Language and Communication*, 23(1), 1–13. 10.2478/plc-2019-0001

[51] Van de Vijver F. R., & Hambleton R. K. (1996). Translating tests: Some practical guidelines. European Psychologist, 1(2), 89–99. 10.1027/1016-9040.1.2.89

[52] Yerlikaya E. (2003). A study on the adaptation of humor styles questionnaire. Unpublished Master Thesis, Adana: Cukurova University Institute of Social Sciences.

[53] Gosling S. D., Rentfrow P. J., & Swann W. B. Jr. (2003). A very brief measure of the Big Five personality domains. *Journal of Research in Personality*, 37, 504–528. 10.1016/S0092-6566(03)00046-1

[54] Atak H. (2013). The Turkish adaptation of the ten-item personality inventory. N*öro Psikiyatri Arşivi*, 50(4), 312–319. doi: 10.4274/npa.y6128 28360563 PMC5363422

[55] Watson D., Clark L. A., & Tellegen A. (1988). Development and validation of brief measures of positive and negative affect: the PANAS scales. *Journal of Personality and Social Psychology*, 54(6), 1063–1070. doi: 10.1037//0022-3514.54.6.1063 3397865

[56] Gençöz T. (2000). Positive and Negative Affect Schedule: A study of validity and reliability. *Türk Psikoloji Dergisi*, 15(46), 19–28.

[57] Rosenberg M. (1979). *Conceiving the Self*. New York, NY: Basic Books.

[58] Tukuş L. (2010). *Benlik Saygısı Değerlendirme Ölçeği-Kısa Formu [The Self Esteem Rating Scale-Short Form]*. *Unpublished Dissertation*. Kocaeli University, Faculty of Medicine, Kocaeli.

[59] Kim H-Y. (2013). Statistical notes for clinical researchers: Assessing normal distribution (2) using skewness and kurtosis. *Restorative Dentistry and Endodontics* 38, 52–54. doi: 10.5395/rde.2013.38.1.52 23495371 PMC3591587

[60] Hoyle R. H., & Smith G. T. (1994). Formulating clinical research hypotheses as structural equation models: A conceptual overview. *Journal of Consulting and Clinical Psychology*, 62(3), 429. doi: 10.1037//0022-006x.62.3.429 8063970

[61] Ungar S. (1984). Self‐mockery: An alternative form of self‐presentation. *Symbolic Interaction*, 7(1), 121–133.

[62] Tsukawaki R., & Imura T. (2020). The light and dark side of self-directed humor: The development and initial validation of the Dual Self-Directed Humor Scale (DSDHS). *Personality and Individual Differences*, 157, doi: 10.1016/j.paid.2020.109835

[63] MacCallum R. C., Roznowski M., & Necowitz L. B. (1992). Model modifications in covariance structure analysis: The problem of capitalization on chance. *Psychological Bulletin*, 111, 490–504. doi: 10.1037/0033-2909.111.3.490 16250105

[64] Bentler P. M. (1990). Comparative fit indexes in structural models. Psychological Bulletin, 107, 238–246. doi: 10.1037/0033-2909.107.2.238 2320703

[65] Bentler P. M., & Satorra A. (2010). Testing model nesting and equivalence. *Psychological Methods*, 15(2), 111–123. doi: 10.1037/a0019625 20515234 PMC2929578

[66] Rothermich K., Ogunlana A., & Jaworska N. (2021). Change in humor and sarcasm use based on anxiety and depression symptom severity during the COVID-19 pandemic. *Journal of Psychiatric Research*, 140, 95–100. doi: 10.1016/j.jpsychires.2021.05.027 34102518 PMC8675002

[67] Mendiburo‐Seguel A., Páez D., & Martínez‐Sánchez F. (2015). Humor styles and personality: A meta‐analysis of the relation between humor styles and the Big Five personality traits. *Scandinavian Journal of Psychology*, 56 *(*3*)*, 335–340. doi: 10.1111/sjop.12209 25786353

[68] Kałowski P., Szymaniak K., & Maciantowicz O. (2021). Exploring the links between trait anger, self-reported sarcasm use, and narcissism. *Advances in Cognitive Psychology*, 17(4), 261–273.

[69] Banasik-Jemielniak N., & Kałowski P. (2022). Socio-cultural and individual factors in verbal irony use and understanding: What we know, what we don’t know, what we want to know. Review of Communication Research, 10.

[70] Tortoriello G. K., Hart W., & Richardson K. (2019). Predicting perceived harmful intent from the Dark Tetrad: A novel cognitive account of interpersonal harm. *Personality and Individual Differences*, 147, 43–52. 10.1016/j.paid.2019.04.020

[71] Blasko D. G., Kazmerski V. A., & Dawood S. S. (2021). Saying what you don’t mean: A cross-cultural study of perceptions of sarcasm. *Canadian Journal of Experimental Psychology*, 75(2), 114–119. doi: 10.1037/cep0000258 34124932

[72] Colston H. L. (2019). Irony as indirectness cross-linguistically: On the scope of generic mechanisms. In: Capone A., García-Carpintero M., & Falzone A. (Eds.). *Indirect reports and pragmatics in the world languages* (pp. 109–131). Springer

