# Supplementary material for: Sarcasm use in Turkish: The roles of personality, age, gender, and self-esteem
Source: PLoS One. 2022 Nov 10;17(11):e0276073. doi: 10.1371/journal.pone.0276073 (PMC9648740; doi:10.1371/journal.pone.0276073)
Supplement: S1 Table — (DOCX) [file pone.0276073.s001.docx]

S1 Table

|  | Skewness | | Kurtosis |
| --- | --- | --- | --- |
| Age in years | | .548 | -.552 |
| Self-esteem | | -.820 | .154 |
| PANAS Positive Affect | | -.302 | -.262 |
| PANAS Negative Affect | | .736 | .222 |
| Self-presentation (self-promotion) | | .245 | .330 |
| Self-presentation (self-depreciation) | | .492 | .905 |
| Sarcasm | | .592 | -.077 |
