# Supplementary material for: Sarcasm use in Turkish: The roles of personality, age, gender, and self-esteem
Source: PLoS One. 2022 Nov 10;17(11):e0276073. doi: 10.1371/journal.pone.0276073 (PMC9648740; doi:10.1371/journal.pone.0276073)
Supplement: S2 Table — (DOCX) [file pone.0276073.s002.docx]

S2 Table

|  |  |  | **coefficient** | **p-value** |
| --- | --- | --- | --- | --- |
| Positive Affect | <--- | Self-esteem | .467 | *** |
| Negative Affect | <--- | Self-esteem | -.434 | *** |
| Positive Affect | <--- | Age | .039 | ,431 |
| Negative Affect | <--- | Age | -.033 | ,519 |
| Positive Affect | <--- | Gender | -.086 | ,077 |
| Negative Affect | <--- | Gender | -.006 | ,900 |
| Self-presentation (self-promotion) | <--- | Positive Affect | .181 | *** |
| Self-presentation (self-depreciation) | <--- | Negative Affect | .144 | ,007 |
| Self-presentation (self-promotion) | <--- | Negative Affect | .172 | ,002 |
| Self-presentation (self-depreciation) | <--- | Positive Affect | -.181 | *** |
| Self-presentation (self-promotion) | <--- | Age | -.181 | *** |
| Self-presentation (self-depreciation) | <--- | Age | -.154 | ,004 |
| Self-presentation (self-promotion) | <--- | Gender | -.049 | ,355 |
| Self-presentation (self-depreciation) | <--- | Gender | .103 | ,048 |
| Sarcasm | <--- | Self-presentation (self-depreciation) | .142 | ,003 |
| Sarcasm | <--- | Self-presentation (self-promotion) | .348 | *** |
| Sarcasm | <--- | Age | -.317 | *** |
| Sarcasm | <--- | Gender | -.110 | ,018 |

Note: *** p<.001
